# Kinesio Taping vs. Auricular Acupressure for the Personalised Treatment of Primary Dysmenorrhoea: A Pilot Randomized Controlled Trial

**DOI:** 10.3390/jpm11080809

**Published:** 2021-08-19

**Authors:** Elena Mejías-Gil, Elisa María Garrido-Ardila, Jesús Montanero-Fernández, María Jiménez-Palomares, Juan Rodríguez-Mansilla, María Victoria González López-Arza

**Affiliations:** 1 ADOLOR Research Group, Department of Medical-Surgical Therapy, Faculty of Medicine and Health Sciences, Extremadura University, 06006 Badajoz, Spain; elenamejias92@gmail.com (E.M.-G.); mariajp@unex.es (M.J.-P.); mvglez@unex.es (M.V.G.L.-A.); 2 Mathematics Department, Faculty of Medicine and Health Sciences, Extremadura University, 06006 Badajoz, Spain; jmf@unex.es

**Keywords:** dysmenorrhea, kinesio tape, auricular acupressure, pain

## Abstract

Background: Dysmenorrhoea is the medical term for menstrual pain. The World Health Organization estimates that up to 81% of women of childbearing age are affected by this condition, and it is one of the leading causes of absenteeism from work and school among women. Although there are pharmacological treatments available for menstrual-pain relief, they do not respond to all women’s needs. Therefore, there is a need to study and develop non-pharmacological alternatives to broaden the individualised treatment options for dysmenorrhea. There are scarce studies published on non-pharmacological treatments, such as kinesio tape and auricular acupressure for the relief of menstrual pain, but the scientific evidence available suggest that these techniques may be beneficial in addressing this problem. The objective of this pilot study was to assess and compare the effectiveness of kinesio tape and auricular acupressure to decrease pain and drug intake in women with primary dysmenorrhoea. Methods: This was a double-blind randomized clinical controlled trial. The period of study was from September 2017 to August 2018. Women enrolled in the University of Extremadura and who had primary dysmenorrhoea were randomized to five groups: control (n = 23), kinesio tape (n = 23), placebo kinesio tape (n = 23), auricular acupressure (n = 23) and placebo auricular acupressure (n = 22). Measures were taken during the pretreatment phase (at four menstrual cycles), during the post-intervention phase (at four menstrual cycles) and during the follow-up phase (at the first and third menstrual cycles after the treatment was completed). The primary outcome measures were mean pain intensity, maximum pain intensity, number of painful days and dose of drug intake during menstruation, measured with the Visual Analogue Scale. The secondary outcome measures were the length of the cycle, the length of menstruation, the drug intake and the type of drug. Results: In all, 108 participants completed the study. The statistical analysis (MANOVA, ANOVA, t-paired and McNemar tests) showed that kinesio tape and auricular acupressure have a beneficial effect on pain relief (mean pain intensity, *p* < 0.001; maximum pain intensity, *p* < 0.001; number of painful days, *p* = 0.021; dose of drug intake, *p* < 0.001). In addition, once the treatments were withdrawn, the auricular-acupressure group maintained lower scores during the first follow-up cycle (*p* < 0.001). Conclusions: Kinesio tape and auricular acupressure decrease pain and drug intake in women with primary dysmenorrhoea. The changes in the auricular-acupressure group seemed to last longer. The results suggest that these techniques could be used as complementary personalised therapies to the pharmacological treatment and not as a substitution.

## 1. Introduction

Dysmenorrhoea is the medical term for menstrual pain. It is a cramping sensation felt in the supra pubic area of the abdomen and can be accompanied by muscle pain, headache and nausea [1,2]. It is classified as primary or secondary according to the absence or presence of underlying pathologies that trigger the pain respectively [2,3].

The World Health Organization estimates that 81% of the female population can be affected by this condition. However, the percentage can vary in different countries [4]. This implies that more than half of the female population of childbearing age experiences this type of pain at least once a month. It has been observed that dysmenorrhoea has a negative impact on the academic, work, sport and social life of women, who can also see their quality of life affected [2,4,5].

Although there are pharmacological treatments available for menstrual-pain relief, they do not respond to all women’s needs. Allergies to drugs, lack of effectiveness or refusal to use medication (for personal, religious or social reasons) leave part of these women with no effective treatment for their symptoms [2,6,7,8,9,10,11].

Therefore, non-pharmacological treatments which provide person-centred and individualised care, such as those proposed in Physiotherapy and Traditional Chinese Medicine, can be very useful. Many publications have focused their studies on the relief of various types of pain through the use of non-pharmacological therapies, such as kinesio tape, thermotherapy, electrotherapy, massage, acupuncture and auricular acupressure obtaining encouraging results [12,13,14,15,16]. In particular, kinesio taping and auricular acupressure are treatment techniques that have been used for a long time in the management of pain from different causes. However, there is very little scientific evidence of the effectiveness of these techniques for primary dysmenorrhoea. Four studies that analyzed the use of kinesio taping in primary dysmenorrhoea can be found in the literature [17,18,19,20]. Their results suggested an improvement of menstrual pain. Similarly, the three articles that studies the effectiveness of auricular acupressure in primary dysmenorrhoea showed a pain-relief effect [21,22,23]. These results would point towards the possible benefits of the use of these non-pharmacological treatment for the management of dysmenorrhoea. 

The application of kinesio taping is based on its proprioceptive and skin receptor stimulation effects. When applied correctly, it influences muscle tone and can induce muscle relaxation. In addition, part of the analgesic effect of this technique is based on its ability to decrease interstitial pressure [24,25]. This causes a reduction of the stimulus received by nociceptors and normalizes local blood and lymphatic circulation, thus also eliminating mediators of pain and inflammation [24,25]. Moreover, the literature also suggests the use of mechanisms related to the Gate Control theory, whereby a tactile sensory stimulus interferes with the perception of pain intensity [26].

Although the mechanism of action of auricular acupressure is still under study, the research conducted by using functional magnetic resonance imaging (fMRI) and positron emission tomography (PET) has revealed the presence of brain activity in the areas corresponding to the structure represented by the point on which the stimulus is applied [27,28,29]. This stimulus has also been shown to trigger specific responses in brain regions related to pain inhibition and to influence the release of endorphins, melatonin and serotonin [27,28].

The objective of this pilot study was to assess the effectiveness of kinesio taping and auricular acupressure improving pain and decreasing drug intake in women with primary dysmenorrhoea, comparing both treatment approaches between them.

## 2. Materials and Methods

### 2.1. Study Design

This pilot study was a single-blind randomized clinical controlled trial. The study took place within the University of Extremadura (Spain), in an outpatient setting. The period of study was from September 2017 to August 2018. The study protocol was approved by the Bioethical Commission of the University of Extremadura in Spain (registration number: 58/2017). The trial was registered with the ClinicalTrials.gov registry (Study Identifier: NCT04400968). All participants signed a written informed consent. The data were guaranteed to be protected and anonymous. The CONSORT statements were used to conduct and report the trial.

### 2.2. Participants and Procedures

The target population was women enrolled in the University of Extremadura who had primary dysmenorrhoea. Participants were recruited in September 2017. The inclusion criteria were women between 18 and 30 years old affected by primary dysmenorrhoea grade 2 and 3 of Andersch and Milsom classification [30], to have attended gynecologist consultation for a general revision in the last 2 years, to have menstrual pain, to have regular menstrual cycles of 21 to 38 days, and to not have an intrauterine device or to be on oral contraceptive treatment. The exclusion criteria were to have been diagnosed with a condition that could influence menstrual-pain perception and to know or have been previously treated with techniques used in the interventions and pregnancy.

In the first place, an interview was conducted at the begging of the academic year (2017/2018) in order to select the sample. Once the participants were recruited, they were assigned an alphanumeric identifier and randomized into the five study groups, using the SPSS statistical program. The program selected the participants to be included in each group equally. Since the sample was not fully divisible among the five groups, the order of preference to complete the groups was allocated by randomly assigning in a ranking from 1 to 5.

Then a pretreatment phase of 4 menstrual cycles started. During this period, the participants completed the questionnaires at their home to collect information regarding the symptoms experienced in each menstrual cycle. The questionnaires were codified by an identifier number assigned to each participant to ensure masking of identity and group allocation and, therefore, to ensure blinding of the data-collection process and analysis. 

Once the pretreatment phase was completed, a four menstrual cycles treatment phase commenced. During this phase the participants continued with the same protocol to collect data and received the treatments assigned to their group. After each cycle finished, the treatments were discontinued, and the follow-up phases started. In the first and second follow-up phase, the data corresponding to the first and the third cycle after the treatments were finished was collected respectively. 

Due to the nature of the treatments, participants knew whether they belonged to one of the kinesio-taping or auricular-acupressure groups. However, they did not know whether the technique applied was the placebo or the real one. The therapist in charge of applying the treatments could not be blinded in order to apply the treatments correctly.

The primary outcome measures were four in total: Mean pain intensity for the 3 first days of menstruation, maximum pain intensity, number of painful days and dose of drug intake. Pain intensity was measured with the Visual Analogue Scale (VAS). This scale measure pain in a scale from 0 to 10, where 0 means no pain and 10 means maximum and excruciating pain [31]. All outcome measures were registered at the initial interview and every day during the bleeding period in the pretreatment, treatment and follow-up phases. As the pretreatment and the treatment phases consisted of four periods each, there were ten measures in all.

The secondary outcome measures included the length of the cycle, the length of menstruation, the drug intake and the type of drug. The epidemiological data of the sample (age, body height, body weight, age of menarche and age of first pain) were collected at the baseline measurement.

**Figure 1:**
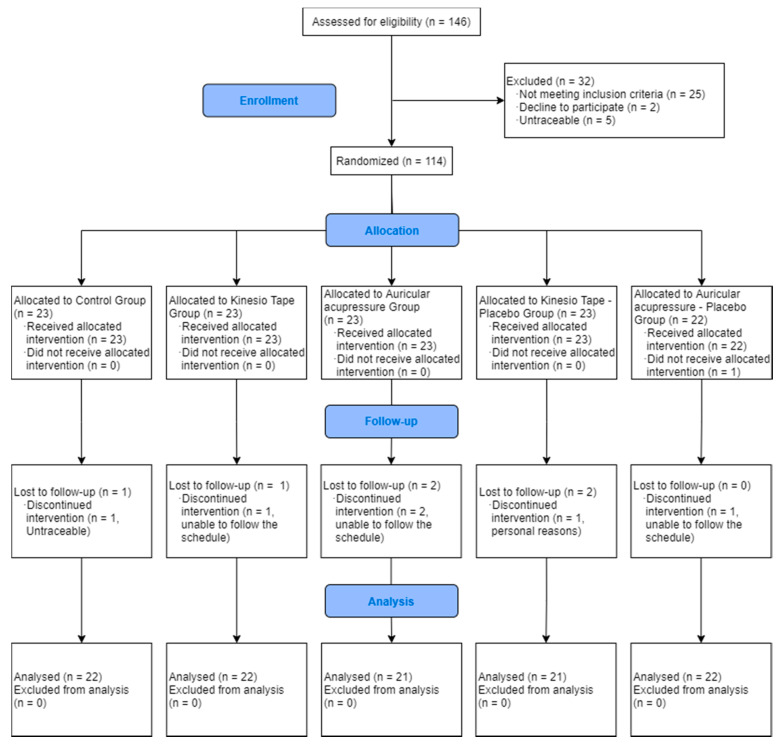
CONSORT flow diagram.

The sample consisted of 114 participants who were randomly allocated to a control group, a kinesio-tape group, a placebo kinesio-tape group, an auricular-acupressure group and a placebo auricular-acupressure group (Figure 1). Details of the intervention following the Template for Intervention Description and Replication (TIDieR) [32] guidelines are provided in Supplemental Materials Table S1.

The therapy and the placebo treatments were always placed within 4 h from the beginning of the menstrual cycle and were maintained during 72 h. When the adhesive tapes lost fixation and became detached, the treatment material was replaced as soon as possible (never later than 2 h from detachment). The cases that could not receive the treatments on the scheduled time were excluded. 

The participants received the necessary information in order to correctly maintain the tapes and adhesives. All groups received the same information, not differentiating between treatment and placebo groups, to ensure blinding. All participants committed to maintain secret their experiences and not to comment with the rest of women during the study period. 

As it was considered unethical, the drug intake was not forbidden if pain relief was necessary. However, all subjects were asked to delay the intake until the appearance of symptoms. In order to control the influence of the medication, the questionnaires included sections where the intake was registered. 

The control group did not receive any treatment. However, the controls completed all the questionnaires to collect the information regarding their symptoms in order to observe their progress with no intervention.

### 2.3. Statistical Analysis

The obtained data were analyzed through the IBM SPSS Statistics 22.0 version (Statistical Package for the Social Sciences). A descriptive analysis of all the outcome measures was performed. Changes on drug intake were analyzed by the McNemar test. In order to assess the primary outcome measures, we distinguished between two steps. Firstly, for each single outcome we applied a one-way multivariate ANOVA considering the ten measurements and the five groups, so that we could contrast the existence of global influence of the treatment. Moreover, drug intake was included as a second factor in the model in order to detect a possible bias due to this circumstance. In the following step, we focused on analysing just the average between the four periods considered during the pretreatment and during treatment stages. This way, the number of phases got reduced to four (average pretreatment, average treatment, follow-up 1 and follow-up 2) for each primary outcome measures. Then, a more exhaustive analysis was performed for each one. On the one hand, we assessed the inter-group differences at each phase by a one-way ANOVA F test or Kruskal–Wallis’s H test (depending on the level of skewness or symmetry of the distribution of the different outcomes). On the other hand, we analyzed separately the within-group changes of the outcome measures during the intervention as compared with the pretreatment scores by a t-paired test or Wilcoxon’s W test.

The sample size does not respond to a previous calculation. However, as many participants as possible were first recruited and posteriorly randomly allocated into five equal groups of 25 women. Finally, according to Cohen’s d, the groups sample size lead to a statistical power of 80% for a 2-sided level 5% *t*-test to detect an effect size of 0.8.

## 3. Results

Figure 1 shows the study flow diagram. Except for the auricular-acupressure placebo group, which had no loses, all groups had similar drop-outs rates. This was 5.6% of the total initial sample.

The average duration of the first cycle was 30.26 ± 0.31 days, with 5.63 ± 1.05 days of menstruation. These values hardly changed along the ten menstruations that were followed up. All the participants reported regular drug intake for pain relief. In fact, 88% of women took medication during the first period previous to the application of the treatments. Nevertheless, we observed that this proportion decreased along the treatment phase to 62% during the last menstruation (*p* < 0.001), since 33 participants stopped taking medication. Most of them (namely 29) belonged to the auricular-acupressure and the kinesio-tape groups. This percentage increased progressively after the treatment during the follow-up phases.

Table 1 and Table 2 include descriptive statistics of the rest of secondary outcomes and the primary ones, respectively.

A one-way multivariate ANOVA showed the overall changes and influence of the treatment for all the primary outcome measures (*p* < 0.001 in the four of them). When including drug intake as the secondary factor in the model, neither interaction (*p* > 0.05) nor drug intake (*p* < 0.05) was significant. Therefore, there was no evidence of bias due to drug intake. Table 2 also includes the evolution of the primary outcome measures during the study in the following way: between-group comparisons were performed by a one way ANOVA F test or by Kruskal–Wallis’s H test, and their *p*-values are shown in the table. Post hoc results are expressed by letters, as usual. Pretreatment within-group comparisons were performed by t-paired or Wilcoxon test. A mean value is marked in bold if it implies a significant improvement in relation to both pretreatment (within-group) and the control group, the placebo auricular-acupressure group and the placebo kinesio-tape group (between-group). 

From the analysis of Table 2, firstly, it can be observed that baseline values of the four primary outcome measures were similar for the five experimental groups (second column of Table 2), as expected. Secondly, the most remarkable is the fact that the auricular-acupressure group achieved the best results in mean, both during the treatment and follow-up one phases, for all the primary outcome measures (mean pain intensity, maximum pain intensity, number of painful days and dose of drug intake). In a deeper analysis and taking into account within-group comparisons, these results showed significant improvements in comparison with the pretreatment phase. Regarding the between-group comparison, we observed that, for the mean pain and maximum pain intensity, the auricular-acupressure group achieved significant improvements during both phases (treatment and follow-up one) in relation to the rest of the groups. In the kinesio-tape group, the results obtained from the treatment phase were similar to those from the auricular-acupressure group. During the first follow-up phase, the auricular-acupressure group performed significantly better. 

In addition, we can also say that, regarding the dose of drug intake, the auricular-acupressure and kinesio-tape groups performed significantly better than the control group and both placebo groups. With regard to the number of painful days, the auricular-acupressure group achieved results significantly better than the control group at the treatment phase and significantly better than the kinesio-tape group at the first follow-up phase.

## 4. Discussion

This pilot study contributes to the scientific literature with the evidence of the effectiveness of two non-pharmacological treatment approaches, kinesio tape and auricular acupressure for the individualised and person-centred management of primary dysmenorrhoea. In addition, it shows, for the first time, a comparative assessment of both techniques.

Our results add new data from a sample of over 100 women to the few studies previously conducted that analyze the effectiveness of these techniques. In addition, the pre- and post-treatment comparisons’ accuracy of our study was improved by the extended period of time used to observe the symptoms in the pretreatment phase. Another strength that of the present clinical trial is the presence of a control group and a placebo group for each treatment technique. Participants were blind at all times in relation to the group they were included in (treatment or placebo) and during the data collection. 

Only four previous studies that applied kinesio tape for menstrual-pain relief were found in the literature [17,18,19,20]. In relation to the application of auricular acupressure in this condition, three studies were the result of the literature review [21,22,23]. In comparison to these studies, it can be observed that our study has a longer period of intervention.

Our results regarding the mean and the maximum pain-intensity levels in the kinesio-tape group and the auricular-acupressure group coincide with the improvements found by different authors that applied these techniques [17,18,19,20,21,22,23]. We were not able to contrast our results in relation to the number of painful days, days of the menstrual cycle and days of the menstruation, as there were no studies found in the literature that analyzed those variables. Although there were no statistically significant changes in the number of days of the menstrual cycle and menstruation after the treatments, we consider that the observation of these variables is important. This is because these variables are risk factors for dysmenorrhoea and can influence the results of the interventions [4].

The scientific evidence available on other non-pharmacological therapies show a great diversity of techniques used for the management of primary dysmenorrhoea, especially related to the field of Physiotherapy and Traditional Chinese Medicine. These include, for example, thermotherapy, massage therapy, electrotherapy, spinal manipulation, Kegel exercises, acupuncture and moxibustion in all its forms (acupressure, electro-acupuncture, laser acupuncture, etc.) [33,34]. However, as in the specific cases of kinesio taping and auricular acupressure, the number of studies published that analyze the effectiveness of these non-pharmacological techniques applied to primary dysmenorrhoea is low. All of these techniques show encouraging results, but, in general, there has been little research on their application to menstrual pain [2,15,33,34,35,36,37,38,39]. A remarkable advantage of kinesio taping and auricular acupressure over the other non-pharmacological techniques mentioned is that they are low-cost techniques, self-applicable after proper training and simple and quick to apply. In addition, the patient can keep the tape or the seeds on and continue with her daily routine, without having to travel to a clinic or invest too much time and money and, therefore, without affecting her rhythm of life.

Although we found no evidence of bias in the pilot study in relation to drug intake, we are not in a position to assess the opposite. Indeed, since most participants took medication during the study, the sample size was big enough for this ambitious statistical task. Nearly 10% of the participants stopped taking medication during all the treatment phase. The dose of drug intake was significantly reduced in the kinesio-tape group (kinesio tape–control = −0.9; kinesio tape–kinesio-tape placebo = −0.81), as well as in the auricular acupressure group (auricular acupressure–control = −1.19; auricular acupressure–auricular ac-pressure placebo = −1.36). The study conducted by Tomás-Rodríguez et al. in 2015 is the only study found that analyzed the drug intake. They assessed the between-group differences and found a difference of 1,09 drug units between the kinesio tape and the placebo groups. Asher et al., in 2010 [40], also found that there are studies on auricular acupressure that have shown a decrease on drug intake for pain relief in other conditions, such as surgery or chronic pain. 

This decrease in drug consumption coincides with the improvement of pain levels and suggests that both treatment approaches could be beneficial for the management of primary dysmenorrhoea. 

The comparisons between the kinesio-tape and the auricular-acupressure groups did not show statistically significant differences during the treatment phase. Nevertheless, when comparing these groups during the follow-up phases, the auricular-acupressure group maintained the improvements achieved during a longer period. These results could not be contrasted, as our study is the first one comparing these treatment techniques. When auricular acupressure has been used for the treatment of other conditions, such as menstrual headache, the results have revealed a reduction of plasma arginine vasopressin and prostaglandine F2α [41]. The most recent research suggests that the cause of primary dysmenorrhoea is the excess of endometrial prostaglandins E2 and F2α, which increase uterine contractions and painful sensation [1]. The longer duration of pain relief maintained by the auricular-acupressure group could be justified by the decrease of prostaglandins F2α and the secretion of pain-inhibiting agents that, according to Wu et al. (2007) [22] and Alimi et al. (2002) [28], this technique achieves.

The results of the present pilot study can have important implications in the clinical practice. Our data show that a kinesio taping and auricular acupressure decrease pain and drug intake in women with dysmenorrhoea. They are two techniques that are increasingly used by practitioners these days and can be performed safely with the appropriate training. This condition, and, in particular, the pain that is associated with it, has an important negative impact on the quality of life of women [2,5] which could be minimized with the use of the treatment approaches described in this study, as a complement to their pharmacological treatment. 

### Study Limitations

The main limitation of our study was the impossibility to blind the therapist that applied the techniques. Due to the nature of the treatment, they could clearly see which group the participant was allocated to.

Furthermore, we consider that it was important to analyze whether the drug intake and the type of medicine used for pain relief could influence these results. In order to assess this possible effect, a two-way multivariate ANOVA, considering as factors drug intake and type of drug, was carried out for each outcome measure. Although there were no significant results, neither for interaction nor for main effects, we cannot dismiss the possible influence. We do not have enough evidence to make a fair decision, since the number of participants that did not take any medicine was so small. In order to analyze the problem at that level, we consider that a bigger sample size would be needed.

## 5. Conclusions

Based on the results obtained in this pilot study, we can conclude that kinesio taping and auricular acupressure have a beneficial effect on pain relief in women with primary dysmenorrhoea. Although both groups showed similar improvements, the changes on the auricular-acupressure group seemed to last longer. The pain relief obtained by both treatment approaches suggests that these techniques could be used as complementary personalised therapies to the pharmacological treatment and not as a substitution. The participants of the kinesio taping and the auricular-acupressure group experienced a decrease in drug intake. 

## Figures and Tables

**Table 1:** Epidemiological data and secondary outcome measures.

	CG (n = 22)	KT (n = 22)	KT-P (n = 21)	AP (n = 21)	AP-P (n = 22)
Height (m)	1.63 ± 0.04	1.61 ± 0.05	1.62 ± 0.06	1.61 ± 0.04	1.60 ± 0.04
Weight (Kg)	56.59 ± 6.44	55.69 ± 6.35	54.53 ± 4.78	54.03 ± 6.23	54.54 ± 5.55
Age (years)	20.91 ± 1.26	20.64 ± 1.05	20.95 ± 1.32	20.95 ± 1.85	21.14 ± 0.99
AM (years)	12.14 ± 1.17	11.86 ± 1.45	12.57 ± 1.12	11.81 ± 1.28	12.64 ± 1.33
AFP (years)	13.68 ± 1.32	14.14 ± 1.75	13.57 ± 1.25	13.48 ± 1.63	13.86 ± 1.12
DC (days)	29.04 ± 1.73	30.09 ± 2.68	29.71 ± 3.01	29.09 ± 1.99	30.36 ± 2.57
DM (days)	5.45 ± 1.22	5.22 ± 0.75	5.52 ± 0.67	5.14 ± 1.42	5.27 ± 0.88

CG, control group; KT, kinesio-tape group; KT-P, kinesio-tape placebo group; AP, auricular-acupressure group; AP-P, auricular-acupressure placebo group; AM, age of menarche; AFP, age of first menstrual pain; DC, duration of the cycles; DM, duration of menstruation.

**Table 2:** Changes (mean ± SD) of the primary outcome measures by groups along the four phases. SD: standard deviation.

Mean Pain Intensity				
	**P-T**	**T**	**FU-1**	**FU-2**
CG	4.85 ± 1.13	4.62 ± 1.02 ^a^	4.93 ± 0.95 ^a^	4.93 ± 0.82
KT	5.18 ± 0.95	**3.40 ± 1.04 ^b^**	4.59 ± 1.18 ^a^	5.24 ± 1.07
KT-P	4.62 ± 1.11	4.63 ± 1.00 ^a^	4.52 ± 0.91 ^a^	4.58 ± 0.95
AP	4.94 ± 0.88	**3.19 ± 1.18 ^b^**	**3.28 ± 1.18 ^b^**	4.74 ± 1.15
AP-P	4.63 ± 1.12	4.60 ± 0.85 ^a^	4.45 ± 1.08 ^a^	4.68 ± 0.88
*F test*	*p* = 0.373	*p* < 0.001	*p* < 0.001	*p* = 0.204
**Maximum pain intensity**				
	**P-T**	**T**	**FU-1**	**FU-2**
CG	7.60 ± 0.70	7.65 ± 0.72 ^a^	7.86 ± 1.03 ^a^	7.72 ± 0.82
KT	7.62 ± 1.02	**6.09 ± 1.44 ^b^**	7.00 ± 1.11 ^a^	7.81 ± 0.95
KT-P	7.78 ± 1.14	7.85 ± 1.02 ^a^	7.57 ± 1.02 ^a^	7.66 ± 1.01
AP	7.34 ± 0.96	**5.83 ± 1.39 ^b^**	**5.66 ± 1.68 ^b^**	7.38 ± 1.20
AP-P	7.61 ± 0.94	7.61 ± 0.84 ^a^	7.59 ± 0.90 ^a^	7.72 ± 0.82
*F test*	*p* = 0.687	*p* < 0.001	*p* < 0.001	*p* = 0.644
**Painful days**				
	**P-T**	**T**	**FU-1**	**FU-2**
CG	3.11 ± 0.97	3.05 ± 0.93 ^a^	3.09 ± 0.87 ^a.b^	3.09 ± 0.81
KT	3.56 ± 0.88	2.63 ± 0.74 ^a.b^	3.27 ± 0.77 ^a^	3.45 ± 0.67
KT-P	3.03 ± 1.00	2.98 ± 0.93 ^a.b^	3.14 ± 0.91 ^a.b^	3.10 ± 1.04
AP	3.34 ± 0.78	2.37 ± 0.57 ^b^	2.48 ± 0.68 ^b^	3.19 ± 0.60
AP-P	3.00 ± 0.83	2.98 ± 0.60 ^a.b^	3.00 ± 0.87 ^a.b^	2.95 ± 0.72
*F test*	*p* = 0.195	*p* = 0.021	*p* = 0.024	*p* = 0.298
**Dose of drug intake**				
	**P-T**	**T**	**FU-1**	**FU-2**
CG	1.78 ± 1.02	1.76 ± 1.04 ^a^	1.68 ± 1.04 ^a^	1.6 ± 0.90
KT	1.84 ± 1.26	**0.89 ± 1.12 ^b^**	1.59 ± 0.96 ^a^	1.72 ± 1.03
KT-P	1.69 ± 1.18	1.61 ± 1.10 ^a^	1.62 ± 1.07 ^a.b^	1.52 ± 0.87
AP	1.94 ± 1.54	**0.60 ± 0.58 ^b^**	0.90 ± 1.51 ^b^	1.62 ± 1.47
AP-P	1.95 ± 1.04	1.83 ± 0.91^a^	1.73 ± 0.94 ^a^	1.77 ± 0.87
*H test*	*p* = 0.847	*p* < 0.001	*p* = 0.009	*p* = 0.864

P-T, pretreatment phase; T, treatment phase; FU-1, follow-up 1 phase; FU-2, follow-up 2 phase; CG, control group; KT, kinesio-tape group; KT-P, kinesio-tape placebo group; AP, auricular-acupressure group; AP-P, auricular-acupressure placebo group. Post-hoc results are expressed by letters as follow: for each column, there are significant differences between groups whose letter is different.

## Data Availability

The data underlying this article cannot be shared publicly in order to maintain the privacy of individuals that participated in the study. The data will be shared upon reasonable request to the corresponding author.

## Supplementary Materials

The following are available online at https://www.mdpi.com/article/10.3390/jpm11080809/s1. Table S1: Descriptions of the interventions conducted following the Template for Intervention Description and Replication (TIDieR).
