# Supplementary material for: Kinesio Taping vs. Auricular Acupressure for the Personalised Treatment of Primary Dysmenorrhoea: A Pilot Randomized Controlled Trial
Source: J Pers Med. 2021 Aug 19;11(8):809. doi: 10.3390/jpm11080809 (PMC8398516; doi:10.3390/jpm11080809)
Supplement: Supplementary file 1 [file jpm-11-00809-s001.zip › jpm-1334986-SI.pdf]

## SUPPLEMENTAL MATERIAL

**Table S1. Descriptions of the interventions conducted following the Template for Intervention Description and Replication (TIDieR)**

| Item nº | Item |
|---|---|
| | **Kinesio tape group** |
| | **BRIEF NAME** |
| **1.** | Kinesio taping for dysmenorrhoea. |
| | **WHY** |
| **2.** | Kinesio tape was used in order to improve menstrual pain in women with dysmenorrhoea.<br>Kinesio taping is a simple, fast and non-invasive technique that is mainly used in the treatment of muscular or joint injuries and pain. It is widely used due to its analgesic, proprioceptive and muscle tone regulating effects (1-3).<br>Studies on the use of kinesio taping in primary dysmenorrhoea suggest that kinesio taping could be an effective technique in the management of menstrual pain (4-7). |
| | **WHAT** |
| **3.** | **Materials:** TEMTEX® Kinesiology Tape (http://temtex.es/), scissors. |
| **4.** | **Procedures:**<br>The kinesio tape group received an intervention that consisted in the standard application of three elastic bandages. Before the tape application, hair was removed from the skin surface when needed and the area cleansed. The tapes were applied at 25% of tension and were placed horizontally covering the area between the anterosuperior iliac spines and the posterosuperior iliac spines and vertically from the navel to the symphysis pubis. All bandages were adhered to the skin with a technique type I with space augmentation. The centre of the tape was removed and applied at 25% of tension. Afterwards, the rest of the protector were removed and the lateral anchors of the tape were adhered with no tension. The participant maintained a slight extension of the trunk while the tape was applied on the anterior aspect of the truck and a slight flexion while the tape was applied in the posterior aspect of the trunk. |
| | **WHO PROVIDED** |
| **5.** | A qualified physiotherapist provided the treatment. She was a member of the Spanish Chartered Society of Physiotherapists and was trained in kinesio taping with certification and wide experience. |
| | **HOW** |
| **6.** | All the treatments were provided individually and face-to-face. |
| | **WHERE** |
| **7.** | The location were the treatment sessions were carried out were the Physiotherapy laboratory classrooms of the University. |
| | **WHEN and HOW MUCH** |

| | |
|---|---|
| **8.** | The kinesio tape was always placed within 4h from the beginning of the menstrual cycle and were maintained during 72h. The treatment was conducted during 4 menstrual cycles. |
| | **TAILORING** |
| **9.** | Not applicable |
| | **MODIFICATIONS** |
| **10.** | Not applicable |
| | **HOW WELL** |
| **11.** | Intervention adherence and attendance at treatment sessions was recorded on an attendance control sheet by the professional guiding the treatment. |
| **12.‡** | Intervention adherence was assessed. Adherence was high, with losses accounting for only 4.3% of participants in this group. |
| **Item nº** | **Item** |
| | **Kinesio tape placebo group** |
| | **BRIEF NAME** |
| **1.** | Placebo Kinesio taping for dysmenorrhoea. |
| | **WHY** |
| **2.** | The aim of the application of this technique was to control the possible placebo effects of the treatment with the real kinesio taping technique for the reduction of menstrual pain in women with primary dysmenorrhoea. |
| | **WHAT** |
| **3.** | **Materials:** TEMTEX® Kinesiology Tape (http://temtex.es/), scissors. |
| **4.** | **Procedures:** |
| | The kinesio tape placebo group had the application of three elastic bandages that were shorter than those used in the kinesio tape group. In addition, the tape was adhered with no tension and not placed in the specific treatment area. |
| | **WHO PROVIDED** |
| **5.** | A qualified physiotherapist provided the treatment. She was a member of the Spanish Chartered Society of Physiotherapists and was trained in kinesio taping with certification and wide experience. |
| | **HOW** |
| **6.** | All the treatments were provided individually and face-to-face. |
| | **WHERE** |
| **7.** | The location were the treatment sessions were carried out were the Physiotherapy laboratory classrooms of the University. |
| | **WHEN and HOW MUCH** |
| **8.** | The placebo kinesio tape was always placed within 4h from the beginning of the menstrual cycle and were maintained during 72h. The placebo treatment was conducted during 4 menstrual cycles. |
| | **TAILORING** |
| **9.** | Not applicable |

| | |
|---|---|
| | **MODIFICATIONS** |
| **10.** | Not applicable |
| | **HOW WELL** |
| **11.** | Intervention adherence and attendance at treatment sessions was recorded on an attendance control sheet by the professional guiding the treatment. |
| **12.ǂ** | Intervention adherence was assessed. Adherence was high, with losses accounting for only 8.7% of participants in this group. |
| **Item nº** | **Item** |
| | **Auricular acupressure group** |
| | **BRIEF NAME** |
| **1.** | Auricular acupressure for dysmenorrhoea. |
| | **WHY** |
| **2.** | The aim of the application of auricular acupressure was to study its effects on the reduction of menstrual pain in women with primary dysmenorrhoea.<br>Auricular acupressure is a non-invasive technique which is easy and quick to apply. It is used for the relief of pain of various origins, although it can be used to treat other symptoms and conditions (8, 9). Research on its efficacy in the treatment of primary dysmenorrhoea suggests that it may be an effective technique in the management of this condition (10-12). |
| | **WHAT** |
| **3.** | **Materials:** Retractable 250 gr. pressure palpator (Sedatelec®), vaccaria seeds (NOVASAN), swabs, ethyl alcohol and tweezers. |
| **4.** | **Procedures:**<br>The auricular acupressure group received an intervention based on the bilateral application of 7 adhesive tapes with vaccaria seeds. The points were located with the retractable pressure palpator. The auricular acupressure points were selected following the protocol for dysmenorrhoea described by Oleson (1). These points were (the name of the European cartography is specified by the name of the point): Shenmen [FT2], uterus [FT5], sympathetic [HI4], kidney [CS6], heart [CI4], endocrine [IT2] and thalamus [PC2]. |
| | **WHO PROVIDED** |
| **5.** | An experienced health professional trained in acupunctural techniques applied the vaccaria seeds. He was a member of the Chartered Society of Physiotherapist and qaulified with the University Specialist Degree in Acupuncture and Moxibusiton from the University of Extremadura. The treatments were also prescribed and supervised by qualified Traditional Chinese Medicine trained acupuncturist (ACMAS Superior School of Acupuncture of Seville and Beijing University) member of the Chartered Society of Physicians of Badajoz (Spain). She also had 22 years of experience and was professor in the University of Extremadura Acupuncture and Moxibustion Postgraduate Course. |
| | **HOW** |
| **6.** | All the treatments were provided individually and face-to-face. |
| | **WHERE** |
| **7.** | The location were the treatment sessions were carried out were the Physiotherapy laboratory classrooms of the University. |

| | |
|---|---|
| | **WHEN and HOW MUCH** |
| **8.** | The auricular acupressure was always placed within 4h from the beginning of the menstrual cycle and were maintained during 72h. The treatment was conducted during 4 menstrual cycles. |
| | **TAILORING** |
| **9.** | Not applicable |
| | **MODIFICATIONS** |
| **10.** | Not applicable |
| | **HOW WELL** |
| **11.** | Intervention adherence and attendance at treatment sessions was recorded on an attendance control sheet by the professional guiding the treatment. |
| **12.[‡]** | Intervention adherence was assessed. Adherence was high, with losses accounting for only 8.7% of participants in this group. |
| **Item nº** | **Item** |
| | **Auricular acupressure placebo group** |
| | **BRIEF NAME** |
| **1.** | Placebo auricular acupressure. |
| | **WHY** |
| **2.** | The aim of the application of this technique was to control the possible placebo effects of the treatment with the real auricular acupressure technique for the reduction of menstrual pain in women with primary dysmenorrhoea. |
| | **WHAT** |
| **3.** | **Materials:** Retractable 250 gr. pressure palpator (Sedatelec®), vaccaria seeds patches (NOVASAN) without the seeds, swabs, ethyl alcohol and tweezers. |
| **4.** | **Procedures:** The auricular acupressure placebo group had adhesive tapes without seeds displaced from the treatment points. |
| | **WHO PROVIDED** |
| **5.** | An experienced health professional trained in acupunctural techniques applied the vaccaria seeds. He was a member of the Chartered Society of Physiotherapist and qualified with the University Specialist Degree in Acupuncture and Moxibusiton from the University of Extremadura. The treatments were also prescribed and supervised by qualified Traditional Chinese Medicine trained acupuncturist (ACMAS Superior School of Acupuncture of Seville and Beijing University) member of the Chartered Society of Physicians of Badajoz (Spain). She also had 22 years of experience and was professor in the University of Extremadura Acupuncture and Moxibustion Postgraduate Course. |
| | **HOW** |
| **6.** | All the treatments were provided individually and face-to-face. |
| | **WHERE** |
| **7.** | The location were the treatment sessions were carried out were the Physiotherapy laboratory classrooms of the University. |

| | |
|---|---|
| | **WHEN and HOW MUCH** |
| **8.** | The placebo auricular acupressure was always placed within 4h from the beginning of the menstrual cycle and were maintained during 72h. The treatment was conducted during 4 menstrual cycles. |
| | **TAILORING** |
| **9.** | Not applicable |
| | **MODIFICATIONS** |
| **10.** | Not applicable |
| | **HOW WELL** |
| **11.** | Intervention adherence and attendance at treatment sessions was recorded on an attendance control sheet by the professional guiding the treatment. . |
| **12.‡** | Intervention adherence was assessed. Adherence in this group was the highest of all groups as there were no losses. |

## REFERENCES


1. Rodriguez-Palencia J. Manual de vendaje neuromuscular: Aplicaciones musculares. Madrid: Bubok. 2013.

2. Kahanov L. Kinesio taping, Part 1: An overview of its use in athletes. Athletic Therapy Today. 2007; 12: 17-18.

3. Kneeshaw D. Shoulder taping in the clinical setting. Journal of Bodywork and Movement Therapies. 2002; 6: 2-8.

4. Yum KS, Kang SG, Han HJ. The effect of balance taping for prevention of menstrual pain in female middle school students. J Phys Ther Sci. 2017; 29(5): 813-818.

5. Tomás-Rodríguez MI, Palazón-Bru A, Martínez-St John DR, Toledo-Marhuenda JV, Asensio-García Mdel R, Gil-Guillén VF. Effectiveness of medical taping concept in primary dysmenorrhea: a two-armed randomized trial. Sci Rep. 2015; 5: 16671.

6. Wefers C, Pijnappel HFJ, Stolwijk NM. Effect of CureTape on menstrual pain in women with primary dysmenorrhoea. [He effect van CureTape op pijn tijdens de menstruatie bij patiënten met primaire dysmenorrhoe]. Ned Tijdschr Fysiother. 2009; 119(6): 193-197.

7. Lim C, Park Y, Bae Y. The effect of the kinesio taping and spiral taping on menstrual pain and premenstrual syndrome. J Phys Ther Sci. 2013; 25(7):761-4.

8. Murakami M, Fox L, Dijkers MP. Ear Acupuncture for Immediate Pain Relief-A Systematic Review and Meta-Analysis of Randomized Controlled Trials. Pain Med. 2017; 18(3): 551-64.

9. Oleson T. Auriculoterapia. Sistemas chino y occidental de acupuntura auricular. 3ª ed. Buenos Aires: Panamericana; 2017.

10. Yeh ML, Hung YL, Chen HH, Wang YJ. Auricular acupressure for pain relief in adolescents with dysmenorrhea: a placebo-controlled study. J Altern Complement Med. 2013; 19(4): 313-8.

11. Wu RD, Zhang HD, Lin LF. [Observation on ear point taping and pressing therapy for treatment of primary dysmenorrhea]. [Article in Chinese] Zhongguo Zhen Jiu. 2007 Nov;27(11):815-7.

12. Cha NH, Sok SR. Effects of Auricular Acupressure Therapy on Primary Dysmenorrhea for Female High School Students in South Korea. J Nurs Scholarsh. 2016; 48(5): 508-16.
